# A case of stridor due to subglottic stenosis resulting from relapsing polychondritis in a young woman

**DOI:** 10.7196/AJTCCM.2018.v24i4.222

**Published:** 2018-12-20

**Authors:** V Gnanasoundran, K K Santhoshraj, M N Prakash

**Affiliations:** 1 Department of Chest and Tuberculosis, Vinayaka Mission’s Medical College, Vinayaka Mission’s Research Foundation (deemed to be University), Karaikal, India; 2 Department of Medicine, Vinayaka Mission’s Medical College, Vinayaka Mission’s Research Foundation (deemed to be University), Karaikal, India

**Keywords:** relapsing polychondritis, stridor, respiratory emergency

## Abstract

Relapsing polychondritis is a rare and severe progressive, multisystem autoimmune disease that affects cartilaginous tissues predominantly of the ear, nose, respiratory system and joints. The rarity of the disease, its unknown aetiology, the array of many possible presenting symptoms, lack of specific investigation for diagnosis and episodic nature, often cause a significant delay in diagnosis. We report a case of a young woman presenting with stridor due to subglottic stenosis of the trachea. In this case, identification of symptoms associated with the stridor facilitated the diagnosis of relapsing polychondritis and initiation of appropriate treatment.

## Background

The condition currently known as relapsing polychondritis (RP) was first identified by Rudolf Jaksch von Wartenhorst in 1923. In 1960, Pearson and his colleagues renamed it from the original polychondropathia to RP, to emphasise the episodic course of the disease. RP is a relatively rare disease, which affects multiple systems, and is characterised by repeated episodes of inflammation and deterioration of cartilage. The exact mechanism is not known, but it is thought to be related to an immune-mediated attack on particular proteins abundant in cartilage. RP affects cartilage of predominantly the ears, nose, larynx and tracheobronchial tree.

## Case history

A 26-year-old female patient, diagnosed with bronchial asthma 2 years earlier and receiving regular treatment, presented to the emergency ward with complaints of breathlessness and fever that had persisted for a day. A detailed history revealed that the patient had developed foot pain at the age of 20 years, which had lasted 6 - 7 months. By the end of that period, she developed joint pain, associated with swelling in both knees and her hands, which lasted about 5 months. She subsequently developed redness of the eyes, which was successfully treated with topical antibiotic drops prescribed by an ophthalmologist. Swelling of the left ear developed 6 months later, with subsequent shrinking of the ear and depression of the nasal bridge. At this stage (after a period of approximately 2 years), she presented with episodes of wheezing, which were considered a result of bronchial asthma and were treated accordingly. There was no family history of similar episodes of joint pain or deformities.

Preliminary examination showed the patient to experience stridor. She was tachypnoeic, tachycardic and hypoxic, with bilateral wheezing in all lung fields. She was given an intravenous injection of hydrocortisone immediately, after which the stridor and breathlessness subsided and her oxygen saturation improved. At the time of this examination, the patient presented with deformed ears, saddle nose-deformity and chest-wall asymmetry. Spine examination revealed kyphoscoliosis. There was no joint tenderness or swelling and no tenderness was present in the laryngeal cartilages. The patient’s hearing was normal.

A blood examination showed the patient to have neutrophilic leukocytosis, with a total count of 12 000 cells/µL. Tests for antinuclear antibodies, antineutrophil cytoplasmic antibodies and rheumatoid factor were negative, as was a slit-skin smear for acid-fast bacilli. Both liver and renal function were normal. A chest X-ray (posteroanterior view) was normal. A computed tomography scan of the neck and thorax showed subglottic stenosis, but no other abnormality was observed in the lung parenchyma. The patient did not consent to a lung function test. A cardiac evaluation was normal. Ophthalmological examination revealed early lattice stromal dystrophy.

An immunologist’s opinion suggested a diagnosis of chondritis involving the left ear, nasal bridge and chest wall. An otorhinolaryngologist’s opinion was sought regarding elective tracheostomy. The patient was clinically diagnosed with RP based on two sets of previously established criteria.^[1,2]^ The initial set of criteria defined that three of the following six clinical features had to be observed for diagnosis: bilateral auricular chondritis; nonerosive seronegative inflammatory polyarthritis; nasal chondritis; ocular inflammation; respiratory tract chondritis, and audiovestibular damage. In 1979, Damiani and Levine^[2]^ reviewed and modified the criteria set out earlier, whereby one of the following three requirements has to be met for a positive diagnosis:

- three of the originally defined criteria satisfied
- one of the originally defined criteria satisfied, together with positive histology results
- two of the originally defined criteria satisfied, together with therapeutic response to corticosteroid or dapsone therapy.

Our patient satisfied both the original and revised criteria^[1,2]^ and was started on 15 mg oral prednisolone daily and 15 mg methotrexate weekly, as advised by a consulting immunologist.

An elective tracheostomy to relieve subglottic stenosis was performed. Immunosuppressant therapy has contributed to the patient’s improvement, which further supports this rare diagnosis.

## Discussion

Life-threatening respiratory symptoms seen in emergency wards could be a manifestation of a rare disease. The diagnosis of RP is based largely on clinical features and the use of laboratory and imaging investigations is purely supportive to rule out other related or associated systemic illnesses.

The onset of RP typically occurs in the fifth decade of life, with most patients between the age of 44 and 51 years at the time of diagnosis; however, cases with either an earlier or a later onset have been reported.^[3]^ Disease onset occurred at the age of 20 in our patient, although she presented with respiratory complications only at the age of 26. Between 20% and 50% of patients may develop respiratory symptoms due to laryngotracheal involvement, which can be a major cause of morbidity and mortality. Respiratory symptoms can be the presenting symptom in 50% of these patients.^[4]^ Owing to inflammation of cartilage in the larynx and tracheobronchial tree, the patient may experience pain and tenderness over the laryngeal cartilage and trachea, hoarseness of voice, a non-productive cough, dyspnoea, stridor or wheezing. Stridor and wheezing were the presenting symptoms in our patient.

Destruction of the thyroid cartilage, acute upper airway collapse and obstruction necessitating emergency tracheostomy are typical later complications. Chronic inflammation may lead to strictures, which are common in the subglottic region. Costochondral cartilage tenderness with dislocation or flail chest can occur. The effectiveness of a cough in clearing secretions is reduced owing to airway collapse and inflammation. Respiratory complications, mostly tracheal collapse and infections, are the leading cause of mortality in RP patients and are reported to vary between 10% and 50%.^[5]^ Overall survival rates from an earlier study have been reported as 74% at 5 years and 55% at 10 years.^[5]^

Life-threatening organ diseases as a result of RP are treated mainly with glucocorticosteroids, whereas less severe symptoms can be treated with non-steroidal anti-inflammatory agents. Inhalational steroids can be used if an obstructive component is seen. Although our patient presented with an obstructive component, she could not be given inhalational steroids as she developed a severe cough on nebulisation. In patients unresponsive or intolerant to steroids, or for steroid-sparing therapy, immunosuppressants such as methotrexate, azathioprine, cyclosporine and chlorambucil may be used. Trentham and Le^[3]^ observed that methotrexate was the most effective non-steroidal drug for symptomatic benefit and for reducing the steroid requirement at an average dose of 17.5 mg/week. Patients with tracheal stricture or collapse may require stenting or tracheal dilatation by interventional bronchoscopy. Silicon T-tubes are an effective treatment for maintaining airway patency in patients with tracheal stenosis. Our patient declined the offer of stenting.

Owing to the autoimmune theory of the pathogenesis of RP, immunomodulatory agents offer an important alternative treatment. Although there are many case reports of satisfactory response to biologic therapy in RP, there are limited data from clinical trials. Standard management cannot be established owing to the rarity of the disease. Treatment of RP is therefore symptomatic and should be tailored to each patient individually based on disease activity and severity.
